# Size and distribution of the global volume of surgery in 2012

**DOI:** 10.2471/BLT.15.159293

**Published:** 2016-03-01

**Authors:** Thomas G Weiser, Alex B Haynes, George Molina, Stuart R Lipsitz, Micaela M Esquivel, Tarsicio Uribe-Leitz, Rui Fu, Tej Azad, Tiffany E Chao, William R Berry, Atul A Gawande

**Affiliations:** a Stanford University Medical Center, Department of Surgery, 300 Pasteur Drive (S067), Stanford, CA 94305, United States of America (USA).; b Ariadne Labs, Brigham and Women's Hospital and Harvard TH Chan School of Public Health, Boston, USA.; c Stanford University Management Science and Engineering, Stanford, USA.; d Stanford University School of Medicine, Stanford, USA.; e Department of Surgery, Massachusetts General Hospital, Boston, USA.

## Abstract

**Objective:**

To estimate global surgical volume in 2012 and compare it with estimates from 2004.

**Methods:**

For the 194 Member States of the World Health Organization, we searched PubMed for studies and contacted key informants for reports on surgical volumes between 2005 and 2012. We obtained data on population and total health expenditure per capita for 2012 and categorized Member States as very-low, low, middle and high expenditure. Data on caesarean delivery were obtained from validated statistical reports. For Member States without recorded surgical data, we estimated volumes by multiple imputation using data on total health expenditure. We estimated caesarean deliveries as a proportion of all surgery.

**Findings:**

We identified 66 Member States reporting surgical data. We estimated that 312.9 million operations (95% confidence interval, CI: 266.2–359.5) took place in 2012, an increase from the 2004 estimate of 226.4 million operations. Only 6.3% (95% CI: 1.7–22.9) and 23.1% (95% CI: 14.8–36.7) of operations took place in very-low- and low-expenditure Member States representing 36.8% (2573 million people) and 34.2% (2393 million people) of the global population of 7001 million people, respectively. Caesarean deliveries comprised 29.6% (5.8/19.6 million operations; 95% CI: 9.7–91.7) of the total surgical volume in very-low-expenditure Member States, but only 2.7% (5.1/187.0 million operations; 95% CI: 2.2–3.4) in high-expenditure Member States.

**Conclusion:**

Surgical volume is large and growing, with caesarean delivery comprising nearly a third of operations in most resource-poor settings. Nonetheless, there remains disparity in the provision of surgical services globally.

## Résumé

**Objectif:**

Estimer le volume mondial d'interventions chirurgicales pratiquées en 2012 et le comparer aux estimations de 2004.

**Méthodes:**

Nous avons recherché sur PubMed des études concernant les 194 États membres de l'Organisation mondiale de la Santé et avons contacté des informateurs clés afin de nous procurer les rapports sur les volumes d'interventions chirurgicales entre 2005 et 2012. Nous avons obtenu des données sur la population et les dépenses totales de santé par habitant pour 2012 et avons caractérisé les États membres selon que ces dépenses étaient très faibles, faibles, moyennes ou élevées. Des rapports statistiques validés nous ont fourni des données sur les césariennes. Pour les États membres qui ne disposaient pas de données archivées sur les interventions chirurgicales, nous avons estimé les volumes au moyen de plusieurs imputations à partir des données sur les dépenses totales de santé. Nous avons estimé le nombre de césariennes en fonction du nombre total d'interventions.

**Résultats:**

Nous avons identifié 66 États membres communiquant des données sur les interventions chirurgicales. Nous avons estimé que 312,9 millions d'opérations (intervalle de confiance de 95%, IC 95%: 266,2–359,5) avaient eu lieu en 2012, soit une augmentation par rapport à l'estimation 2004 de 226,4 millions d'opérations. Seules 6,3% (IC 95%: 1,7–22,9) et 23,1% (IC 95%: 14,8–36,7) des opérations ont eu lieu dans des États membres aux dépenses très faibles et faibles, ce qui représente respectivement 36,8% (2573 millions de personnes) et 34,2% (2393 millions de personnes) de la population mondiale de 7001 millions de personnes. Les césariennes représentaient 29,6% (5,8/19,6 millions d'opérations; IC 95%: 9,7–91,7) du volume total d'interventions chirurgicales pratiquées dans les États membres aux dépenses très faibles, mais seulement 2,7% (5,1/187,0 millions d'opérations; IC 95%: 2,2–3,4) dans les États membres aux dépenses élevées.

**Conclusion:**

Le volume d'interventions chirurgicales est important et ne cesse d'augmenter. Les césariennes représentent près d'un tiers des opérations dans les pays aux plus faibles ressources. Néanmoins, la fourniture de services chirurgicaux dans le monde continue de présenter des disparités.

## Resumen

**Objetivo:**

Estimar el volumen global de intervenciones quirúrgicas en 2012 y compararlo con las estimaciones de 2004.

**Métodos:**

Se realizaron búsquedas de estudios en PubMed y se contactó a informantes clave para obtener información sobre el volumen de intervenciones quirúrgicas entre 2005 y 2012 para los 194 Estados Miembros de la Organización Mundial de la Salud. Se obtuvieron datos sobre la población y el gasto total en salud per cápita en 2012 y se categorizó a los Estados Miembros por gasto muy bajo, bajo, medio y elevado. Se obtuvieron datos acerca del número de cesáreas de informes estadísticos validados. Para los Estados Miembros sin datos quirúrgicos registrados, se estimaron los volúmenes por varias asignaciones utilizando datos sobre el gasto total en salud. Se estimó el numero de cesareas como un porcentaje del total de las intervenciones quirúrgicas.

**Resultados:**

Se identificaron 66 Estados Miembros que registran datos quirúrgicos. Se estimó que en 2012 se realizaron 312,9 millones de operaciones (intervalo de confianza del 95%, IC del 95%: 266,2–359,5), lo que significa un aumento de la estimación de 226,4 millones de operaciones de 2004. Únicamente un 6,3% (IC del 95%: 1,7–22,9) y un 23,1% (IC del 95%: 14,8–36,7) de las operaciones fueron realizadas en Estados Miembros de gasto muy bajo y bajo, lo que representa el 36,8% (2 573 millones de personas) y el 34,2% (2 393 millones de personas) de la población mundial de 7 001 millones de personas, respectivamente. Las cesáreas abarcaron el 29,6% (5,8/19,6 millones de operaciones; IC del 95%: 9,7–91,7) del total del volumen de intervenciones quirúrgicas en Estados Miembros con un gasto muy bajo, pero únicamente el 2,7% (5,1/187,0 millones de operaciones; IC del 95%: 2,2–3,4) en Estados Miembros con un gasto elevado.

**Conclusión:**

El volumen de intervenciones quirúrgicas es cada vez mayor, y las cesáreas abarcan casi un tercio de las operaciones en los lugares con menos recursos. No obstante, sigue habiendo una diferencia en el suministro de servicios quirúrgicos a nivel global.

## ملخص

الغرض تقدير عدد العمليات الجراحية التي تم إجراؤها في عام 2012 على مستوى العالم ومقارنته بالتقديرات التي تم التوصل إليها في عام 2004.

الطريقة بحثنا في محرك البحث PubMed عن بعض الدراسات واتصلنا بالمبلّغين الرئيسيين بشأن التقارير المتعلقة بأعداد العمليات الجراحية التي تم إجراؤها في الفترة بين عامي 2005 و2012، وذلك فيما يتعلق بالدول الأعضاء في منظمة الصحة العالمية البالغ عددها 194 دولة. وقد حصلنا على بيانات بشأن السكان وإجمالي النفقات الصحية للفرد الواحد لعام 2012 وصنفنا الدول الأعضاء في شرائح النفقات الشديدة الانخفاض، والنفقات المنخفضة، والنفقات المتوسطة، والنفقات المرتفعة. وتم الحصول على البيانات المتعلقة بالولادة القيصرية من التقارير الإحصائية الموثقة. وفيما يتعلق بالدول الأعضاء التي لا تتوفر بها بيانات مسجلة عن العمليات الجراحية، فقد أجرينا تقديرًا للأعداد بالاستعانة بطريقة حساب القيم التعويضية المتعددة باستخدام البيانات المتعلقة بالنفقات الصحية. واتخذنا الولادات القيصرية كنسبة لتقدير جميع العمليات الجراحية.

النتائج حددنا 66 دولة من بين الدول الأعضاء يتوفر بها تقارير ببيانات العمليات الجراحية. وحسب تقديرنا تم إجراء 312.9 مليون عملية جراحية (بنسبة أرجحية مقدارها 95‏%: 266.2 – 359.5) في عام 2012، بزيادة عن الأعداد المقدَّرة في عام 2004 تبلغ 226.4 مليون عملية جراحية. بنسبة 6.3‏% فقط (بنسبة أرجحية مقدارها 95‏%: 1.7 – 22.9) و23.1‏% (بنسبة أرجحية مقدارها 95‏%: 14.8 – 36.7) من العمليات الجراحية التي تم إجراؤها في الدول الأعضاء من شريحتي النفقات شديدة الانخفاض والنفقات المنخفضة بما يمثل 36.8‏% (2573 مليون شخص) و34.2‏% (2393 مليون شخص) من التعداد العالمي للسكان الذي يبلغ 7001 مليون نسمة، على التوالي. وشكلت الولادات القيصرية 29.6%‏ (5.8/19.6 مليون عملية جراحية؛ بنسبة أرجحية مقدارها 95‏%: 9.7 – 61.7) من إجمالي عدد العمليات الجراحية في الدول الأعضاء من شريحة النفقات الشديدة الانخفاض، ولكن بلغت النسبة 2.7‏% فقط (5.1/187.0 مليون عملية جراحية؛ بنسبة أرجحية مقدارها 95‏%: 2.2 – 3.4) في الدول الأعضاء من شريحة النفقات المرتفعة.

الاستنتاج إن عدد العمليات الجراحية كبير ومتزايد، وتمثل الولادة القيصرية ما يقرب من ثلث العمليات الجراحية في معظم المواقع التي تفتقر إلى الموارد. وبالرغم من ذلك، يظل الفارق في توفير الخدمات الجراحية قائمًا على المستوى العالمي.

## 摘要

**目的:**

旨在评估 2012 年全球外科手术总量,并将其与 2004 年数据进行比较。

**方法:**

对于世界卫生组织的 194 个成员国,我们搜索 PubMed 进行调查,并联系关键受访者,获得 2005 年至 2012 年间外科手术总量的报告。我们获得 2012 年人口数据及人均卫生开支总额的数据,并将成员国分为开支非常低、低、中等及高这四类。利用经过验证的统计报告获知剖腹产手术的数据。对于没有外科手术记录数据的成员国,我们通过使用卫生开支总额数据进行的多次估算,推算出外科手术总量。我们也将剖腹产手术作为一种外科手术进行评估。

**结果:**

我们确定 66 个成员国报告了外科手术数据。我们估计 2012 年外科手术总量为 312,900,000(95% 置信区间,95% CI:266.2–359.5),相较于 2004 年,增长 226,400,000 次手术。其中,仅 6.3% (95% CI:1.7–22.9) 和 23.1% (95% CI:外科手术发生于开支非常低和低的成员国,而在全球 7,001,000,000 人口中,其分别占 36.8%(2,573,000,000 人口)和 34.2%(2,393,000,000 人口)。在开支非常低的成员国,剖腹产手术量在外科手术总量中占 29.6%(5,800,000/19,600,000 外科手术量;95% CI:9.7–91.7),但在开支高的成员国,仅占 2.7%(5,100,000/187,000,000 外科手术量;95% CI:2.2–3.4)。

**结论:**

外科手术数量很大,且不断增长,其中在资源匮乏地区,剖腹产手术约占外科手术总量的三分之一。不过,外科手术服务在全球范围内仍有所不同。

## Резюме

**Цель:**

Подсчитать общемировой объем хирургических операций в 2012 г. и сравнить его с результатами оценки, проведенной в 2004 г.

**Методы:**

Для получения результатов для 194 государств-членов Всемирной организации здравоохранения осуществлялся поиск по базе данных PubMed на предмет исследований, а также были запрошены отчеты по объему хирургических операций у ключевых информаторов за период 2005–2012 гг. Были получены данные о численности населения и общих расходах на здравоохранение на душу населения для 2012 г., и государства-члены были разделены на группы: с очень низким уровнем расходов, с низким уровнем расходов, со средним уровнем расходов и с высоким уровнем расходов. Сведения о количестве кесаревых сечений были получены из утвержденных статистических отчетов. Для получения результатов для государств-членов, в которых данные по хирургии не фиксировались, объемы были подсчитаны с помощью нескольких условных значений на основе данных об общих расходах на здравоохранение. Была подсчитана доля кесаревых сечений от всех хирургических операций.

**Результаты:**

66 государств-членов сообщили данные о хирургических операциях. Согласно подсчетам 312,9 млн операций (доверительный интервал 95%, 95% ДИ: 266,2–359,5) было проведено в 2012 г. Этот показатель превышает результат подсчетов 2004 г., составивший 226,4 млн операций. Лишь 6,3% (95% ДИ: 1,7–22,9) и 23,1% (95% ДИ: 14,8–36,7) операций было проведено в государствах-членах с очень низким и низким уровнем расходов, что соотносится с 36,8% (2 573 млн людей) и 34,2% (2 393 млн людей) соответственно от общемирового населения, составляющего 7 001 млн людей. Доля кесаревых сечений составила 29,6% (5,8 из 19,6 млн операций; 95% ДИ: 9,7–91,7) от общего объема хирургических операций в государствах-членах с очень низким уровнем расходов и лишь 2,7% (5,1 из 187,0 млн операций; 95% ДИ: 2,2–3,4) в государствах-членах с высоким уровнем расходов.

**Вывод:**

Объем операций значителен и увеличивается, и доля кесаревых сечений составляет приблизительно треть всех операций в странах, испытывающих острый недостаток ресурсов. Однако при этом в мире сохраняется неравномерное распределение ресурсов, необходимых для хирургических операций.

## Introduction

Surgical care is essential for managing diverse health conditions – such as injuries, obstructed labour, malignancy, infections and cardiovascular disease – and an indispensable component of a functioning health system.^1^^–^^3^ International organizations, including the World Health Organization (WHO) and the World Bank, have highlighted surgery as an important component for global health development.^3^^,^^4^ However, surgical care requires coordination of skilled human resources, specialized supplies and infrastructure.

As low- and middle-income countries expand their economies and basic public health improves, noncommunicable diseases and injuries comprise a growing proportion of the disease burden.^5^ Investments in health-care systems have increased in the last decade, but the effect on surgical capacity is mostly unknown.^6^^,^^7^

Based on modelling of available data, it was estimated that 234.2 million operations were performed worldwide in 2004.^8^ The majority of these procedures took place in high-income countries (58.9%; 138.0 million), despite their relative lower share of the global population.

Here, we estimated the global volume of surgery in 2012. We also estimated the proportion of surgery due to caesarean delivery, since studies done in low-income countries have found that emergency obstetric procedures – especially caesarean deliveries – represent a high proportion of the total surgical volume.^9^^,^^10^

## Methods

### Population and health databases

For the years 2005 to 2012, we obtained population and health data for 194 WHO Member States. These data included total population, life expectancy at birth, percentage of total urban population, gross domestic product (GDP) per capita in United States dollars (US$) and total health expenditure per capita in US$.^6^^,^^11^ For 11 Member States, where certain population or health data were not available from either WHO or the World Bank, we used data from other similar sources.^12^^,^^13^ All US$ were adjusted for inflation to the year 2012, using the consumer price index for general inflation.^14^ For Member States with reported surgical data, we also obtained population and health data from the year for which surgical volume was reported. We classified Member States based on their health spending. Member States spending US$ 0–100 per capita on health were classified as very-low-expenditure Member States (*n* = 50); US $101–400 as low-expenditure Member States (*n* = 54); US$ 401–1000 as middle-expenditure Member States (*n* = 46); and over US$ 1000 as high-expenditure Member States (*n* = 44).^8^

### Surgical data sources

Operations were defined as procedures performed in operating theatres that require general or regional anaesthesia or profound sedation to control pain. We searched PubMed for the most recent annual surgical volume reported after 2004, using each Member State name along with the following keywords and phrases for all WHO Member States: “surgery”, “procedures”, “operations”, “national surgical volume” and “national surgical rate”. Depending on the Member State, we conducted our search in English, French and/or Spanish. To obtain email addresses for ministers or officials working for the ministry of health or individuals responsible for auditing surgical data at a national level, we searched the internet for the websites of ministries of health or national statistical offices. We contacted these persons to request the most recently reported total volume of operations based on the above definition.

From the database of the Organisation for Economic Co-operation and Development (OECD) we obtained surgical volume for 26 countries; 14 of these countries had total surgical volume data as well as detailed data for a subset of procedures (termed a shortlist by OECD), while the other 12 countries only had data for the shortlist.^15^ For the 14 countries, we used both data sets in combination with publicly available data on total health expenditure to define the relationship between the shortlist and the reported total surgical volume. We used this relationship to estimate total surgical volume for the 12 countries that only had shortlist and total health expenditure data. The average relative difference between the observed total surgical rate and extrapolated total surgical rate was 13.7% for these 14 countries; in a leave-one-out cross validation, the relative average bias was 16%.

For the Member States from which we obtained surgical data between 2005 and 2013, we calculated the annual surgical volume per 100 000 population for the year that the data were reported for the Member State by using the total population estimate for the same year.

### Statistical analysis

#### Model development

To develop a predictive model for surgical rates, we first investigated the bivariate Spearman correlations between surgical rate and five a priori country-level variables: total population, life expectancy, percent urbanization, GDP per capita and total health expenditure per capita. We selected total health expenditure per capita as the only explanatory variable based on the results of Spearman correlations. We then did two sensitivity analyses: Spearman partial correlations and a multivariable regression model using the Lasso approach for variable selection.^16^

Our final predictive model contained only total health expenditure per capita. Finally, we log-transformed total health expenditure per capita and surgical rate to account for their right-skewed distribution.

#### Missing data analysis

To determine if any of the five a priori country-level predictors was related to the probability that a country’s surgical rate was missing, we fitted a multivariable logistic regression (Table 1).^17^ This model allowed us to determine variables associated with surgical rate. These variables could then be included in the imputation model to predict the rates for the Member States with missing data. The only variable significantly associated with whether a country’s surgical rate was missing was total health expenditure per capita, which was already included in the imputation model.

**Table 1:** Comparison of Member States of the World Health Organization with or without available surgical volume data, 2012

Characteristic	Member States with surgical data *n* = 66	Member States without surgical data *n* = 128	*P^a^*
**No. of Member States by region (%)**			0.319
African Region	9 (14)	37 (29)	–
Region of the Americas	11 (17)	24 (19)	–
Eastern Mediterranean Region	7 (11)	15 (12)	–
European Region	30 (45)	23 (18)	–
South-East Asian Region	5 (8)	6 (5)	–
Western Pacific Region	4 (6)	23 (18)	–
**Mean population size, in millions (95% CI)**	48.0 (6.4–89.7)	29.9 (9.9–49.9)	0.346
**Mean life expectancy, years (95% CI)**	73.9 (71.7–76.1)	68.5 (66.9–70.1)	0.128
**% of population living in urban areas (95% CI)**	62.9 (57.2–68.5)	53.3 (49.2–57.3)	0.772
**Mean GDP per capita, US$ (95% CI)**	21 745 (15 882–27 608)	10 147 (6 493–13 801)	0.219
**Mean total health expenditure per capita, US$ (95% CI)**	1 887 (1 315–2 460)	616 (408–825)	0.004

CI: confidence interval; GDP: gross domestic product; US$: United States dollars.

^a^ *P* values are derived from a multivariate logistic regression model.

Note: Inconsistencies arise in some values due to rounding.

#### Imputation model

To find the best fitting model for the relation between surgical rate and total per capita health expenditure, we built a spline model, positing splines with zero, one, two or three inflection points.^18^^–^^20^ The best-fitting spline model was selected based on leave-one-out cross-validation, in which the predicted surgical rate value for a country was estimated based on a model that had been fitted after omitting data for that country. We used total per capita health expenditure from 2012 for our imputation model of surgical rates. The Democratic People's Republic of Korea, Somalia and Zimbabwe had no available total health expenditure data for 2012. Since the Pearson correlation between health expenditure in 2012 and any single year between 2000 and 2011 for all other Member States was ≥ 0.97, we extrapolated total health expenditure for these Member States by using their expenditure from previous years. As we did not have reported total health expenditure for 2013, we assumed that surgical rates or volume reported for 2013 were equivalent to 2012 values. For the 25 Member States with surgical data reported before 2012, we extrapolated 2012 estimates for these using a multiple imputation model that treated 2012 surgical rate data as missing for these 25 Member States.

For Member States with missing surgical volume data, we used multiple imputation and our predictive model to arrive at 2012 surgical rate estimates.^21^ We produced 300 imputed data sets to estimate the mean global surgical volume and its corresponding 95% confidence interval. Using the imputed country-level surgical rates and population estimates for 2012 we calculated the number of operations performed in each country in 2012. We also used published caesarean delivery data to calculate the proportion of surgical volume accounted for by caesarean delivery for each country.^22^ These data came primarily from the Global Health Observatory data repository,^23^ World Health Statistics 2010,^24^ the World Health Report 2010,^25^ the Demographic and Health Surveys^26^ and OECD.^15^

To compare the 2004 estimates with the new 2012 estimates, we used the same data on reported surgical rate from 56 countries that we used in the 2004 modelling exercise^8^ and did a spline analysis. We tested spline models with zero, one, two or three inflection points for the 2004 data. The spline model with two inflection points had the highest adjusted cross validation *R^2^*, as with the 2012 data. We evaluated the change in surgical rates that occurred for each health expenditure group between 2004 and 2012. This ensured that any observed changes in estimated volume were not driven by the updated modelling approach (details available from corresponding author).

We used SAS software version 9.2 (SAS Institute Inc., Cary, United States of America) for all statistical analyses. Two-sided statistical tests were done and all *P*-values less than 0.05 were considered statistically significant.

## Results

### Model development

The total health expenditure per capita was the most highly correlated variable with surgical rate (Spearman correlation, *r* = 0.87297; *P* < 0.0001; Table 2; available at: http://www.who.int/bulletin/volumes/94/3/15-159293). The sensitivity analyses showed that after adjusting for total health expenditure per capita, none of the other variables remained significant. WHO regions were also not significantly associated with surgical rate (*P* = 0.09).

**Table 2:** Bivariate Spearman correlations between surgical rate and five a priori country-level variables and Spearman partial correlations adjusting for total health expenditure

Variable	Spearman correlation	*P*	Spearman partial correlation	*P*
Total health expenditure per capita	0.87297	< 0.0001	NA	NA
Life expectancy	0.77536	< 0.0001	−0.06327	0.6166
GDP	0.81359	< 0.0001	−0.24295	0.0512
Urban population	0.69607	< 0.0001	0.00659	0.9585
Population size	−0.18869	0.1292	−0.11665	0.3548

GDP: gross domestic product; NA: not applicable.

**Fig. 1:**
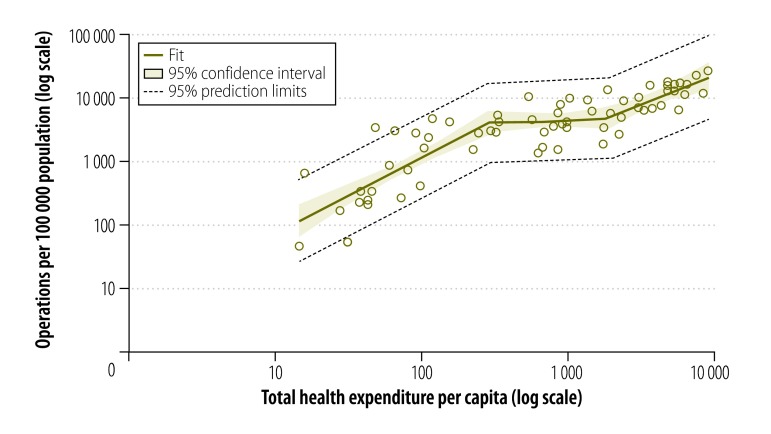
Relationship between observed operations and total health expenditure per capita, 66 Member States of the World Health Organization, 2012

Notes: Total health expenditure adjusted to United States dollars (US$) for the year 2012. Correlation between observed operations and total health expenditure per capita  was *r* = 0.87297 (*P* < 0.0001). The adjusted cross validation was *r^2^* = 0.7449. Inflexion points correspond with adjusted total health expenditure per capita; the first inflexion point is US$ 288 and the second inflection point is US$ 1950.

Fig. 1 shows the best fitting spline model for surgical rate based on total health expenditures, with two inflection points at US$ 288 and US$ 1950 per person per year (*r*^2^: 0.7449). The models with zero, one and three inflection points had adjusted cross validation *r*^2^ of 0.7064, 0.7071 and 0.7332 respectively.

### Surgical volume

We obtained surgical data from 66 Member States (Table 3; available at: http://www.who.int/bulletin/volumes/94/3/15-159293). Using multiple imputation, we extrapolated the volume of surgery for each country without reported surgical data (Table 4; available at: http://www.who.int/bulletin/volumes/94/3/15-159293). For the year 2012, we estimated the total global volume to be 312.9 million operations – an increase of 38.2% from an estimated 226.4 million operations in 2004. The estimated mean global surgical rate was 4469 operations per 100 000 people per year (Table 5).

**Table 3:** Surgical rate and volume for 66 Member States of the World Health Organization with observed surgical data, 2005–2012

Member State (year of reported data)	Population in 2012	Total health expenditure per capita^a^	Annual no. of operations	Annual no of operations per 100 000 population^b^
Afghanistan (2008)^27^	29 824 536	37	61 920	229
Armenia (2012)^c,d^	2 969 081	150	123 861	4 172
Australia (2012)^28^	22 723 900	6 140	2 477 096	10 901
Austria (2012)^c,^^29^	8 429 991	5 407	1 178 284	13 977
Bahrain (2012)^30^	1 317 827	895	51 992	3 945
Bangladesh (2011)^e,^^31^	154 695 368	28	247 178	162
Belgium (2012)^32^	11 128 246	4 711	1 976 833	17 764
Bhutan (2012)^33^	741 822	90	19 954	2 690
Bolivia (Plurinational State of) (2010)^34^	10 496 285	112	228 622	2 251
Bulgaria (2005)^35^	7 305 888	322	398 180	5 145
Burkina Faso (2012)^36^	16 460 141	38	54 379	330
Canada (2012)^c,e,f,g,h^^37^^,^^38^	34 754 312	5 741	2 382 956	6 857
Chad (2012)^39^	12 448 175	31	6593	53
China (2012)^c,^^40^	1 350 695 000	322	39 500 000	2 924
Colombia (2012)^i^	47 704 427	530	5 108 304	10 708
Costa Rica (2012)^41^	4 805 295	951	202 519	4 214
Cuba (2012)^c,^^42^	11 270 957	558	539 528	4 787
Cyprus (2011)^43^	1 128 994	2 168	29 663	2 657
Czech Republic (2012)^c,^^44^	10 510 785	1 432	658 811	6 268
Denmark (2007)^45^	5 591 572	6 321	892 682	16 345
El Salvador (2009)^46^	6 297 394	244	172 972	2 797
Estonia (2012)^47^	1 325 016	1 010	126 883	9 576
Ethiopia (2011)^e,^^48^	91 728 849	14	38 220	43
Finland (2012)^j^	5 413 971	4 232	428 000	7 905
France (2012)^32^	65 676 758	4 690	10 709 393	16 306
Georgia (2012)^c,^^49^	4 490 700	333	189 478	4 219
Germany (2012)^32^	80 425 823	4 683	9 802 610	12 188
Guatemala (2012)^k^	15 082 831	226	231 288	1 533
Hungary (2012)^32^	9 920 362	987	319 718	3 223
Ireland (2012)^32^	4 586 897	3 708	299 335	6 526
Israel (2012)^l^	7 910 500	2 289	400 808	5 067
Italy (2012)^32^	59 539 717	3 032	4 118 831	6 918
Latvia (2011)^50^	2 034 319	843	119 184	5 791
Liberia (2010)^e,^^51^	4 190 435	45	11 502	331
Lithuania (2011)^50^	2 987 773	906	262 270	8 140
Luxembourg (2012)^32^	530 946	7 452	116 452	21 933
Mali (2009)^52^	14 853 572	48	450 260	3 321
Malta (2012)^m^	419 455	1 835	55 501	13 232
Mexico (2012)^n^	120 847 477	618	1 613 405	1 335
Myanmar (2011)^53^	52 797 319	16	337 726	650
Nepal (2011)^54^	27 474 377	42	56 768	209
Netherlands (2012)^32^	16 754 962	5 737	2 787 778	16 639
New Zealand (2012)^c,^^55^	4 433 000	3 292	280 310	6 323
Nicaragua (2010)^56^	5 991 733	118	278 874	4 594
Oman (2012)^57^	3 314 001	690	90 804	2 740
Peru (2011)^58^	29 987 800	289	894 243	3 020
Poland (2012)^32^	38 535 873	854	583 957	1 515
Portugal (2011)^59^	10 514 844	2 350	890 965	8 439
Qatar (2009)^60^	2 050 514	1 762	29 572	1 891
Republic of Korea (2012)^61^	50 004 441	1 703	1 709 706	3 419
Rwanda (2010)^e,^^62^	11 457 801	59	86 041	850
Saudi Arabia (2012)^63^	28 287 855	795	1 002 474	3 544
Sierra Leone (2012)^64^	5 978 727	96	24 152	404
Slovakia (2012)^65^	5 407 579	1 326	475 111	8 786
Slovenia (2012)^32^	2 057 159	1 942	116 009	5 639
Spain (2010)^66^	46 761 264	3 056	4 657 900	10 110
Sri Lanka (2006)^67^	19 858 000	89	579 820	2 920
Sweden (2012)^32^	9 519 374	5 319	1 485 940	15 610
Switzerland (2012)^32^	7 996 861	8 980	2 073 050	25 923
Syrian Arab Republic (2010)^68^	22 399 254	105	339 825	1 578
Turkey (2012)^32^	73 997 128	665	1 223 059	1 653
Uganda (2011)^e,^^48^	36 345 860	42	84 874	241
United Kingdom (2012)^69^	63 695 687	3 647	9 732 653	15 280
United States (2007)^70^	313 873 685	8 895	36 457 210	12 087
Yemen (2012)^71^	23 852 409	71	65 114	273
Zambia (2010)^o^	14 075 099	79	94 145	722

^a^ Adjusted to 2012 United States dollars.

^b^ Surgical rate is calculated using the total population for the year the surgical data were available.

^c^ Surgical data from 2013.

^d^ Data obtained via official communication with Armenian Ministry of Health, Armenia,13 August 2014.

^e^ Regional rates extrapolated to entire country.

^f^ Data obtained via official communication with Office of the Honourable Monica Ell, Nunavut Department of Health. Nunavut, Canada, 30 July 2014.

^g^ Data obtained via official communication with the Saskatchewan Ministry of Health, data obtained from the Surgical Initiative database. Saskatchewan, Canada, 5 August 2014.

^h^ Data obtained via official communication with the Office of Minister Doug Graham, Health and Social Services of Yukon, Canada, 15 August 2014.

^i^ Data obtained via official communication with Dirección de Epidemiología y Demografía, Ministerio de Salud y Protección Social de Colombia, Colombia, 22 August 2014.

^j^ Data obtained from Senior Planning Officer of the Finnish National Institute for Health and Welfare National Institute for Health and Welfare, Finland, 23 July 2014.

^k^ Data obtained via official communication with Ministerio de Salud Pública y Asistencia Social, Sistema de Información Gerencial de Salud – SIGSA. Viceminsterio de Hospitales*,* Guatemala, 10 July 2014.

^l^ Data obtained from Head of Division of Health Information, Israeli Ministry of Health, Israel, 21 August 2014.

^m^ Data obtained via personal communication. Distefano S, National Hospitals Information System, Directorate for Health Information & Research, Malta, 30 July 2014.

^n^ Data obtained via personal communication with Rosas Osuna SR, Sistema Nacional de Información en Salud (SINAIS): Secretaría de Salud*,* Mexico Ministry of Health, Mexico, 12 March 2014.

^o^ Data obtained via personal communication with Bowman K, Children’s Hospital of Wisconsin, United States of America, 17 April 2014

**Table 4:** Average imputed surgical rates and expected yearly number of operations, based on total health expenditure per capita, for 128 Member States of the World Health Organization with missing surgical volume data, 2012

Country	Population in 2012	Total health expenditure per capita^a^	Average imputed no. of operations per 100 000 population per year	Expected range of operations in 2012^b^
Albania	2 801 681	228	4 991	123 393–156 263
Algeria	38 481 705	279	6 663	2 253 295–2 875 033
Andorra	78 360	3 057	9 263	5 980–8 537
Angola	20 820 525	190	4 812	867 905–1 136 052
Antigua and Barbuda	89 069	681	5 210	3 962–5 319
Argentina	41 086 927	995	5 519	1 993 467–2 541 889
Azerbaijan	9 295 784	398	4 225	339 029–446 449
Bahamas	371 960	1 647	7 067	22 715–29 857
Barbados	283 221	938	5 303	13 256–16 779
Belarus	9 464 000	339	4 593	373 612–495 757
Belize	324 060	259	6 199	17 214–22 965
Benin	10 050 702	33	406	35 503–46 076
Bosnia and Herzegovina	3 833 916	447	4 859	158 739–213 844
Botswana	2 003 910	384	4 674	80 047–107 289
Brazil	198 656 019	1 056	6 128	10 500 890–13 844 633
Brunei Darussalam	412 238	939	5 740	20 850–26 472
Burundi	9 849 569	20	217	18 381–24 422
Cabo Verde	494 401	144	2 636	11 225–14 836
Cambodia	14 864 646	51	666	86 263–111 749
Cameroon	21 699 631	59	816	154 105–200 182
Central African Republic	4 525 209	18	165	6 607–8 307
Chile	17 464 814	1 103	5 462	843 337–1 064 491
Comoros	717 503	38	470	2 916–3 826
Congo	4 337 051	100	1 568	60 014–76 016
Cook Islands	10 777	511	4 760	403–623
Côte d'Ivoire	19 839 750	88	1 481	259 012–328 483
Croatia	4 267 558	908	5 798	218 765–276 118
Democratic People's Republic of Korea	24 763 188	76	1 298	276 561–366 155
Democratic Republic of the Congo	65 705 093	15	144	82 327–106 897
Djibouti	859 652	129	2 576	19 458–24 832
Dominica	71 684	392	4 717	2 805–3 959
Dominican Republic	10 276 621	310	4 153	377 226–476 327
Ecuador	15 492 264	361	4 538	610 398–795 822
Egypt	80 721 874	152	2 889	2 066 134–2 598 531
Equatorial Guinea	736 296	1 138	5 834	37 487–48 421
Eritrea	6 130 922	15	147	7 796–10 238
Fiji	874 742	177	3 487	26 874–34 128
Gabon	1 632 572	397	4 471	63 539–82 433
Gambia	1 791 225	26	311	4 715–6 426
Ghana	25 366 462	83	1 338	296 538–382 153
Greece	11 092 771	2 044	5 886	570 323–735 563
Grenada	105 483	478	4 769	4 391–5 669
Guinea	11 451 273	32	384	38 463–49 596
Guinea-Bissau	1 663 558	30	333	4 788–6 289
Guyana	795 369	235	5 771	39 069–52 737
Haiti	10 173 775	53	776	66 467–91 429
Honduras	7 935 846	195	4 198	294 312–372 041
Iceland	320 716	3 872	12 163	33 989–44 026
India	1 236 686 732	61	904	9 801 319–12 556 488
Indonesia	246 864 191	108	1 839	3 957 879–5 120 005
Iran (Islamic Republic of)	76 424 443	490	4 106	2 767 543–3 508 289
Iraq	32 578 209	226	5 409	1 521 217–2 003 067
Jamaica	2 707 805	318	4 337	103 013–131 876
Japan	127 561 489	4 752	14 508	16 388 287–20 626 119
Jordan	6 318 000	388	4 475	248 911–316 588
Kazakhstan	16 791 425	521	4 972	731 544–938 337
Kenya	43 178 141	45	619	232 365–301 898
Kiribati	100 786	187	3 998	3 468–4 591
Kuwait	3 250 496	1 428	5 971	172 105–216 085
Kyrgyzstan	5 607 200	84	1 390	68 768–87 164
Lao People's Democratic Republic	6 645 827	40	508	29 864–37 621
Lebanon	4 424 888	650	5 425	206 805–273 335
Lesotho	2 051 545	138	2 777	50 047–63 910
Libya	6 154 623	578	4 831	260 219–334 448
Madagascar	22 293 914	18	175	34 593–43 541
Malawi	15 906 483	25	297	41 090–53 311
Malaysia	29 239 927	419	4 537	1 177 889–1 475 530
Maldives	338 442	558	5 070	14 551–19 770
Marshall Islands	52 555	590	5 063	2 292–3 030
Mauritania	3 796 141	52	702	23 302–29 963
Mauritius	1 291 167	444	4 493	51 187–64 848
Micronesia (Federal States of)	103 395	405	4 537	4 042–5 340
Monaco	37 579	6 708	20 262	6 563–8 666
Mongolia	2 796 484	232	4 908	120 159–154 342
Montenegro	621 081	493	5 110	27 903–35 568
Morocco	32 521 143	190	3 929	1 104 656–1 450 854
Mozambique	25 203 395	37	496	108 974–141 142
Namibia	2 259 393	473	4 785	92 473–123 729
Nauru	9 378	564	4 674	347–529
Niger	17 157 042	25	293	43 349–57 053
Nigeria	168 833 776	94	1 596	2 360 057–3 028 546
Niue	1 269	1 270	6 365	47–115
Norway	5 018 573	9 055	29 239	1 276 741–1 657 982
Pakistan	179 160 111	34	423	656 418–859 980
Palau	20 754	972	6 552	1 138–1 581
Panama	3 802 281	723	5 194	174 850–220 103
Papua New Guinea	7 167 010	114	2 076	130 103–167 403
Paraguay	6 687 361	392	4 386	253 242–333 423
Philippines	96 706 764	119	2 385	2 005 550–2 607 277
Republic of Moldova	3 559 519	239	5 789	178 368–233 757
Romania	20 076 727	420	5 134	887 449–1 174 096
Russian Federation	143 178 000	887	5 577	6 938 584–9 031 846
Saint Kitts and Nevis	53 584	825	5 492	2 478–3 408
Saint Lucia	180 870	556	4 578	7 266–9 293
Saint Vincent and the Grenadines	109 373	340	4 734	4 303–6 053
Samoa	188 889	245	5 609	9 101–12 087
San Marino	31 247	3 792	11 921	3 222–4 228
Sao Tome and Principe	188 098	109	1 990	3 173–4 311
Senegal	13 726 021	51	715	84 466–111 699
Serbia	7 199 077	561	5 068	316 905–412 754
Seychelles	88 303	521	4 858	3 772–4 806
Singapore	5 312 400	2 426	7 275	335 808–437 171
Solomon Islands	549 598	148	3 016	14 468–18 681
Somalia	10 195 134	20	231	19 986–27 089
South Africa	52 274 945	645	4 991	2 235 713–2 982 830
South Sudan	10 837 527	27	311	29 067–38 266
Sudan	37 195 349	115	2 042	658 712–860 547
Suriname	534 541	521	4 947	22 660–30 230
Swaziland	1 230 985	259	6 176	66 589–85 453
Tajikistan	8 008 990	55	764	53 256–69 118
Thailand	66 785 001	215	4 775	2 756 949–3 621 426
The former Yugoslav Republic of Macedonia	2 105 575	327	4 476	81 800–106 710
Timor-Leste	1 148 958	50	684	6 835–8 892
Togo	6 642 928	41	530	30 889–39 566
Tonga	104 941	238	5 650	5 016–6 842
Trinidad and Tobago	1 337 439	972	5 865	68 535–88 354
Tunisia	10 777 500	297	4 627	420 162–577 232
Turkmenistan	5 172 931	129	2 460	111 503–143 051
Tuvalu	9 860	577	5 017	389–601
Ukraine	45 593 300	293	4 882	1 891 091–2 560 965
United Arab Emirates	9 205 651	1 343	5 891	473 401–611 217
United Republic of Tanzania	47 783 107	41	454	193 051–240 876
Uruguay	3 395 253	1 308	6 256	186 105–238 742
Uzbekistan	29 774 500	105	1 878	492 861–625 376
Vanuatu	247 262	116	2 084	4 480–5 827
Venezuela (Bolivarian Republic of)	29 954 782	593	5 376	1 383 223–1 837 617
Viet Nam	88 772 900	102	1 865	1 459 314–1 852 719
Zimbabwe	13 724 317	228	5 168	620 938–797 504

^a^ Adjusted to 2012 United States dollars.

^b^ Ranges for volume of surgery are derived from the 99% prediction interval from 300 imputed data sets for each country based on total health expenditure per capita.

**Table 5:** Comparative rate and volume of surgery for Member States of the World Health Organization, by total health expenditure group, 2004 and 2012

Variable	Member State total health expenditure group^a^												Global	
	Very low			Low			Middle			High				
	2004	2012		2004	2012		2004	2012		2004	2012		2004	2012
No. of Member States	47	50		60	54		47	46		38	44		192	194
Population, in millions (% of global population)	2248 (34.8)	2573 (36.8)		2258 (35.0)	2393 (34.2)		940 (14.7)	799 (11.4)		1007 (15.6)	1236 (17.7)		6453 (100)	7001 (100)
Mean estimated surgical rate, per 100 000 population per year (95% CI)	394 (273–516)	666 (465–867)		1851 (1162–2540)	3973 (2 320–5625)		3944 (2857–5030)	4822 (3085–6560)		11 629 (9560–13 697)	11 168 (9151–13 186)		3941 (3333–4541)	4469 (3693–5245)
Change in surgical rate, % (95% CI)	–	69.0 (9.9–160.0)		–	114.6 (23.1–274.2)		–	22.3 (−22.2–92.1)		–	−4.0 (−25.4–23.6)		–	–
Estimated no. of surgeries in millions (95% CI)	14.0 (1.8–26.2)	19.6 (7.4–51.7)		41.4 (5.6–77.3)	72.2 (56.7–91.9)		31.9 (19.3–44.5)	34.1 (19.8–58.7)		139.0 (131.5–146.4)	187.0 (155.8–224.5)		226.4 (181.9–270.8)	312.9 (266.2–359.5)
% of global volume of surgery (95% CI)	6.2 (1.9–21.5)	6.3 (1.7–22.9)		18.3 (5.5–63.2)	23.1 (14.8–36.7)		14.1 (7.2–28.5)	10.9 (5.0–24.5)		61.4 (46.5–84.1)	59.8 (41.0–88.8)		100 (NA)	100 (NA)

CI: confidence interval; NA: not applicable; US$: United States dollars.

^a^ Total health expenditure adjusted to US$ for the year 2012. Very low-expenditure Member States were defined as per capita total expenditure on health of US$ 100 or less; low-expenditure Member States as US$ 101–400; middle-expenditure Member States as US$ 401–1000; and high-expenditure Member States as more than US$ 1000.

Note: Inconsistencies arise in some values due to rounding.

The rate of surgery increased significantly for all Member States spending US$ 400 or less per capita in total health expenditures (Table 5). Across the health expenditure brackets, mean estimated surgical rates in 2012 ranged from 666 to 11 168 operations per 100 000 people. Of the total global volume of surgery, 6.3% (19.6/312.9 million operations) was performed in very-low-expenditure Member States which accounted for 36.8% (2.573/7.001 billion people) of the world’s population in 2012, while 59.8% (187.0/312.9 million operations) of the surgical volume took place in the high-expenditure Member States which account for 17.7% (1.236/7.001 billion people) of the world’s population. The biggest increase in the rate of surgery occurred in very-low- and low-expenditure Member States (69.0%; from 394 to 666 operations per 100 000 population per year and 114.6%, from 1851 to 3973 operations per 100 000 population per year, respectively), while middle- and high-expenditure Member States experienced no significant change.

Caesarean delivery data were more widely available than overall surgical data, with data from 172 Member States. In very-low-expenditure settings, caesarean delivery accounted for 29.6% (5.8/19.6 million operations) of all operations performed. However, in high-expenditure Member States this percentage was only 2.7% (5.1/187.0 million operations; Table 6). Worldwide, caesarean deliveries account for nearly one in every 14 operations performed.

**Table 6:** Volume and proportional contribution of caesarean delivery for Member States of the World Health Organization, by total health expenditure group, 2012

Caesarean delivery	Member State health expenditure group^a^				Global
	Very low	Low	Middle	High	
Estimated no. in millions (95% CI)	5.8 (5.8–5.9)	7.8 (7.8–7.9)	4.1 (4.0–4.3)	5.1 (5.0–5.1)	22.9 (22.5–23.2)
% of caesarean deliveries (95% CI)	25.5 (24.9–26.0)	34.2 (33.7–34.8)	18.0 (17.1–19.0)	22.2 (21.9–22.6)	100 (NA)
% of global volume of surgery (95% CI)	29.6 (9.7–91.7)	10.8 (8.2–14.4)	12.1 (6.2–23.5)	2.7 (2.2–3.4)	7.3% (6.1–9.0)

CI: confidence interval; NA: not applicable; US$: United States dollars.

^a^ Total health expenditure adjusted to US$ for the year 2012. Very low-expenditure Member States were defined as per capita total expenditure on health of US$ 100 or less; low-expenditure Member States as US$ 101–400; middle-expenditure Member States as US$ 401–1000; and high-expenditure Member States as more than US$ 1000.

Note: Inconsistencies arise in some values due to rounding.

## Discussion

We estimate 266.2 to 359.5 million operations were performed in 2012. This represents an increase of 38% over the previous eight years. We note the largest increase in operations was in very-low- and low-expenditure Member States. However, about one in every 20 operations globally was done in very-low-expenditure Member States, despite these Member States representing well over one third of the total global population. Comparing very-low-expenditure Member States with high-expenditure Member States, the gap in access is even larger. These disparities may be even larger when examining the distribution of access to surgical care within individual Member States, an undertaking that is beyond the scope of this study.

The proportion of caesarean delivery were higher in Member States with lower surgical volume. This likely demonstrates that obstetrical emergencies are prioritized as a surgical intervention in Member States with scarce resources, but also suggests that other surgical conditions are left poorly attended in these settings. The findings serve to highlight the importance of improving surgical capacity to address both obstetrical and other surgical conditions.

Surgical data were lacking from many Member States. Compared with the data availability for the 2004 estimates, only 10 more Member States now had available data. This contrasted with caesarean delivery data, which were available for the majority of Member States. Given the efforts of the maternal health community and the importance of caesarean delivery in supporting improved maternal outcomes, our findings are not surprising. The challenge of accessing data on surgical care impede the understanding and monitoring of surgery as a component of global health care. Without standardized and accessible data, it is difficult for researchers and policy-makers to contextualize and prioritize surgical access and quality when discussing health system strengthening.

In 2015, the World Health Assembly passed a resolution strengthening emergency and essential surgical care and anaesthesia as a component of universal health coverage.^72^ The increases in injuries and noncommunicable diseases present a challenge for weak health systems already struggling with a high infectious burden of disease.^73^ Not only do injuries and many noncommunicable diseases require surgical intervention, in many resource-poor settings neglected infections – such as typhoid and tuberculosis – are not treated in a timely fashion and therefore require surgical care.^74^

The increase in surgical output in very-low-expenditure Member States over the last eight years suggests that these Member States are placing an increasing importance on access to emergency and essential surgical services. However, the Lancet Commission on Global Surgery has estimated that five billion people lack access to safe, affordable surgical and anaesthesia care when needed and an additional 143 million operations are required to address emergency and essential conditions in low- and middle-income countries.^3^

The lack of standardized surgical data globally is both a limitation of and the reason for undertaking this study. As part of the WHO Safe Surgery Saves Lives programme for which the 2004 estimates of global surgical volume was performed, our group proposed a standard set of metrics for surgical surveillance.^75^ We continued to have difficulty during this study obtaining standardized data regarding surgical intervention. The data were not located or reported in any standardized way and required our research team to compile the information from multiple agencies, ministries, health reports and published literature, as there was no central source for collecting or reporting these data. Some ministry reports may include only state and government facilities and not hospitals run privately or by nongovernmental organizations, which can provide substantial surgical capacity. Thus the volume we report may be an underestimate. Regardless, the non-included facilities are unlikely to close the gap in care between Member States or change our findings. In addition, there was no differentiation between surgical care undertaken in urban versus rural areas. There is likely a large discrepancy in surgical access and provision of surgical care within a single country.

OECD, which had previously collected total operative volume as reported in our last study,^8^ has changed its methods and now reports on only a subset of procedures. Thus our analysis required an additional step to turn these data into comprehensive estimates of volume, adding another layer of uncertainty.

Many of the same limitations of the previous analysis were present here. We focused on operations performed in an operating theatre as these are most likely to involve high complexity, acuity and risk. Our study is thus limited by the manner in which such operations and procedures are recorded. We recognize that many minimally invasive procedures can be undertaken outside an operating theatre, as can many image-guided procedures, thus potentially undercounting what might be considered surgery in these settings. Many minor procedures may also be undertaken in the operating room to improve pain control or exposure or because of availability of resources and equipment, thus creating variability within our count. However, by standardizing our definition, we limited the difficulties associated with the variability in case mix and practice patterns across Member States and settings.

As only one third of Member States reported data on surgical volume, our estimates of overall volume of surgery continue to rely on modelling techniques. We noted changes in the slope of the curve of our spline regression over the range of health expenditure, in particular between the two spline inflections, likely reflecting the heterogeneity of Member States. Furthermore, while the imputation strategy was aimed at a global estimate, the estimate for any particular country may be imprecise. However, our modelling strategy was based on the strong explanatory power of per capita expenditure on health as a determinant of surgical volume. Health expenditure per capita was the only variable that was significantly associated with whether surgical rate data was missing, and multiple imputation protects against systemic bias from data that are missing at random.

## Conclusion

Surgical volume continues to grow, particularly in very-low- and low-expenditure Member States. However, surgical surveillance continues to be weak and poorly standardized and limits the precision of these estimates, yet the systematic evaluation of access, capacity, delivery and safety of care is paramount if surgical services are to support a programme of health system strengthening. Furthermore, the relationship of surgical provision to population health outcomes is not clear, and interventions such as surgery that include substantial risk to patients must be carefully considered. Many patients receive surgical care, yet safety and quality-of-care remain poorly measured and a low priority in many Member States.
